# Methylene Blue Near-Infrared Fluorescence Imaging in Breast Cancer Sentinel Node Biopsy

**DOI:** 10.3390/cancers14071817

**Published:** 2022-04-03

**Authors:** Oliver Budner, Tomasz Cwalinski, Jarosław Skokowski, Luigi Marano, Luca Resca, Natalia Cwalina, Leszek Kalinowski, Richelle Hoveling, Franco Roviello, Karol Polom

**Affiliations:** 1 Frauenheilkunde und Geburtshilfe, HELIOS Kliniken Schwerin, Wissmarsche Street 393-397, 19-055 Schwerin, Germany; oliver.budner@helios-gesundheit.de; 2 Department of Surgical Oncology, Faculty of Medicine, Medical University of Gdansk, Marii Sklodowskiej-Curie Street 3a, 80-217 Gdansk, Poland; cwalinski.tomasz@gmail.com (T.C.); jaroslaw.skokowski@gumed.edu.pl (J.S.); 3 Department of Medical Laboratory Diagnostics–Biobank Fahrenheit BBMRI.pl, Medical University of Gdansk, Debinki Street 7, 80-211 Gdańsk, Poland; lekal@gumed.edu.pl; 4 Biobanking and Biomolecular Resources Research Infrastructure Poland (BBMRI.pl), 80-210 Gdańsk, Poland; 5 Department of Medicine, Surgery and Neurosciences, Unit of General Surgery and Surgical Oncology, University of Siena, Viale Bracci 16, 53-100 Siena, Italy; luigi.marano@unisi.it (L.M.); lucaresca3@gmail.com (L.R.); franco.roviello@unisi.it (F.R.); 6 Department of Pediatrics Ascension, St. John Children’s Hospital, Detroit, MI 48236, USA; ncwalina@gmail.com; 7 BioTechMed/Department of Mechanics of Materials and Structures, Gdansk University of Technology, Gabriela Narutowicza Street 11/12, 80-233 Gdansk, Poland; 8 Quest Medical Imagining, Industrieweg 41, 1775 PW Middenmeer, The Netherlands; richelle.hoveling@quest-innovations.com

**Keywords:** breast cancer, methylene blue, sentinel node biopsy, fluorescence

## Abstract

**Simple Summary:**

Currently the gold standard for sentinel node biopsy in breast cancer patients is radioactive nanocolloid and a blue dye. In the age of fluorescence guided surgery new fluorophores are used and methylene blue presents some fluorescent properties. This study is the first in a clinical series presenting the possible use of methylene blue as a fluorescent dye for the identification of sentinel nodes in breast cancer sentinel node biopsy. We presented a feasibility of this new method and also in additional experiments because of the quenching effect limitation, found possible dilution of methylene blue presenting improved fluorescence.

**Abstract:**

Introduction: Fluorescence-based navigation for breast cancer sentinel node biopsy is a novel method that uses indocyanine green as a fluorophore. However, methylene blue (MB) also has some fluorescent properties. This study is the first in a clinical series presenting the possible use of MB as a fluorescent dye for the identification of sentinel nodes in breast sentinel node biopsy. Material and methods: Forty-nine patients with breast cancer who underwent sentinel node biopsy procedures were enrolled in the study. All patients underwent standard simultaneous injection of nanocolloid and MB. We visualized and assessed the sentinel nodes and the lymphatic channels transcutaneously, with and without fluorescence, and calculated the signal-to-background ratio (SBR). We also analyzed the corresponding fluorescence intensity of various dilutions of MB. Results: In twenty-three patients (46.9%), the location of the sentinel node, or the end of the lymphatic path, was visible transcutaneously. The median SBR for transcutaneous sentinel node location was 1.69 (range 1.66–4.35). Lymphatic channels were visible under fluorescence in 14 patients (28.6%) prior to visualization by the naked eye, with an average SBR of 2.01 (range 1.14–5.6). The sentinel node was visible under fluorescence in 25 patients (51%). The median SBR for sentinel node visualization with MB fluorescence was 2.54 (range 1.34–6.86). Sentinel nodes were visualized faster under fluorescence during sentinel node preparation. Factors associated with the rate of visualization included diabetes (*p* = 0.001), neoadjuvant chemotherapy (*p* = 0.003), and multifocality (*p* = 0.004). The best fluorescence was obtained using 40 μM (0.0128 mg/mL) MB, but we also observed a clinically relevant dilution range between 20 μM (0.0064 mg/mL) and 100 μM (0.032 mg/mL). Conclusions: For the first time, we propose the clinical usage of MB as a fluorophore for fluorescence-guided sentinel node biopsy in breast cancer patients. The quenching effect of the dye may be the reason for its poor detection rate. Our analysis of different concentrations of MB suggests a need for a detailed clinical analysis to highlight the practical usefulness of the dye.

## 1. Introduction

Sentinel node biopsy (SNB) is currently the gold-standard procedure for evaluating the stage of disease in early breast cancer [1,2]. The concept of SNB has a long history, but the first description of the procedure was published by Morton and Cochrane in 1992 in melanoma patients [3]. The first application of blue dye in SNB was proposed by Giuliano et al. for breast cancer in 1994 [4]. The authors identified blue-dye-stained sentinel lymph nodes in 114 of 174 (65.5%) patients. Today’s standard for SNB is a dual technique that includes blue dye and/or radioactive technetium-99m (^99m^Tc)-labelled [5] nanocolloid. Blue dye identifies not only stained nodes but also lymphatic vessels between the injection site and axillary lymph nodes. Three different types of blue dye can be used in SNB: methylene blue (MB), isosulfan blue, and patent blue V. Recently, other dyes have been proposed for use in SNB, with indocyanine green (ICG) representing a suitable fluorophore for this application. In 2005, Kitai et al. proposed fluorescence-guided navigation of SNB in breast cancer could be performed using near-infrared light [6]. Since then, many publications have shown its practical usage and clinical potential [7,8,9]. Near-infrared fluorescence (NIRF) is a technique that uses light in the near-infrared range for the visualization of structures stained by a fluorophore [10]. After excitation by a specific wavelength of near-infrared (NIR) light, the fluorophore can be visualized by a special imaging system that detects the reemitted light of a different length from the excited fluorophore [11,12,13]. Three fluorophores have been approved for SNB NIRF by the Federal Drug Administration and the European Medicines Agency: ICG, MB, and 5-amino-levulinic acid (5-ALA). MB is not considered a pure dye that displays NIR properties [14,15,16].

When used as a fluorophore, MB is excited by light at a wavelength of 668 nm, with an emission of 688 nm, which is detected within the visible light spectrum of 400–700 nm [14]. Few initial reports about the use of MB’s NIRF properties have been published [17,18,19]. MB has been used to visualize the parathyroid glands during surgery, to localize the ureters for the prevention of accidental damage to them during operations, to localize different pancreatic tumors, and in breast cancer margin detection during breast conserving therapy [17,19,20,21,22,23]. A recent publication by Zhang et al. presented the first videos of an SNB using MB as a fluorophore [7].

The aim of the current study was to present, for the first time in a clinical series of patients, the possible use of MB as a fluorescent dye for SNB performed during surgery for breast cancer. We also explored the detection rate of different dilutions of MB and their corresponding fluorescence.

## 2. Methods

In this prospective series of 49 patients, only patients with a diagnosis of invasive breast cancer were included in the study. All patients received care at the Surgical Oncology Department at the University Clinical Center in Gdansk, Poland. An institutional review board approved the study (No. NKBBN/92/2018-2020). The patients signed an informed consent form. Each patient qualified for SNB after a multidisciplinary board review. Certain patients (9) received pre-operative treatment in the form of chemotherapy (6/49; 12.2%) or hormonal therapy (3/49; 6.1%) depending on their qualifications according to current breast cancer treatment guidelines.

All patients underwent the standard sentinel node procedure. MB (METIBLO) in 10 mg/mL vials was purchased from Laboratoires STEROP NV, Scheutlaan, Brussels, Belgium. On the day of surgery, approximately 1–3 h prior to the operation, a dose of 100 MBq 99m technetium nanocolloid was administered at an injection site close to the areola. Directly before the operation, the attending surgeon injected 1 mL of MB, at a standard concentration of 10 mg/mL, at the injection site close to the areola. After the injection of MB, the area was massaged to aid in the assessment of proper lymphatic drainage. After surgical scrubbing and preparation of the operative field with sterile draping, NIR fluorescence imaging was performed using a Quest Spectrum (Quest Medical Imaging, Middenmeer, The Netherlands) fluorescence imaging device after dimming the operative lights. The Quest Spectrum (Quest Medical Imaging, Middenmeer, The Netherlands) is designed and developed for open and minimally invasive image-guided surgery using NIRF imaging. The fluorescence imaging system is designed to visualize two types of fluorescent probes that are not visible to the naked eye: Cy5.5 and ICG. It can also be used with any other probe that has similar fluorescence properties. For this study, the Cy5.5 mode was used to visualize MB. Tissues were illuminated with a wavelength of 680 nm and visualized at approximately 710 nm. During this process, a color image of the surgical field can be visualized simultaneously with NIRF, allowing surgical guidance.

The camera was installed onto a flexible arm. Fluorescence imaging was performed at a distance of 20–30 cm from the surgical field. As darkness is necessary for better visualization of MB’s fluorescence, the surgical field itself was illuminated using the white light source of the camera system. Afterward, a standard inspection by a handheld gamma camera system, Gamma Finder II (W.O.M. World of Medicine GMBH; Berlin, Germany), was performed using the standard SNB protocol. Each surgeon had access to all three images—gamma probe, naked eye for color visualization, and NIR fluorescence imaging—to aid in the detection of the sentinel node (SLN).

**Figure 1:**
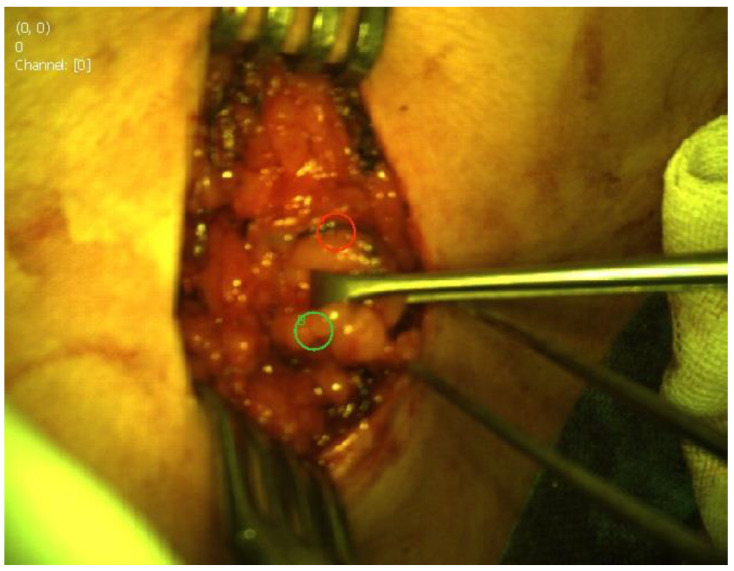
Sentinel node biopsy visible under normal light.

**Figure 2:**
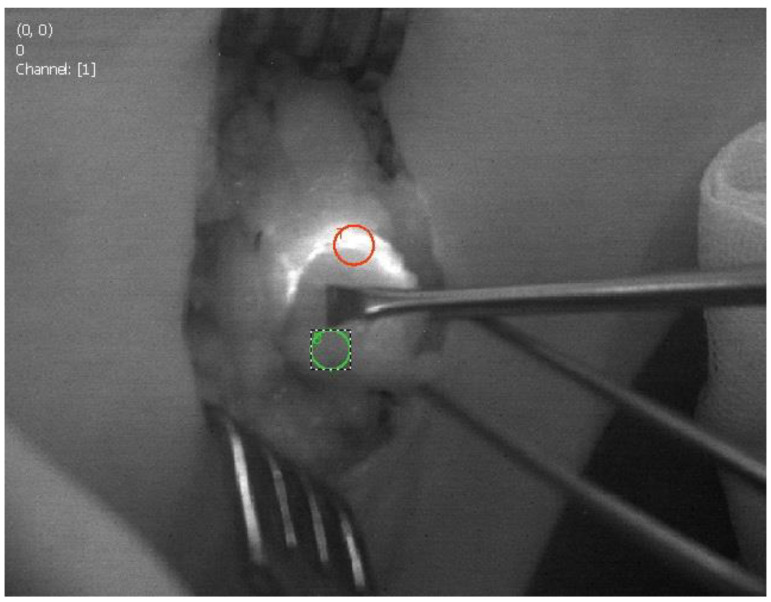
Sentinel node biopsy visible under fluorescence. The ROI are marked.

The possibility of the camera system visualizing fluorescence with “hands free” imaging enabled continuous visualization of the lymphatic vessels and nodes during the surgery (Figure 1 and Figure 2). The visibility of the fluorescence signal of the SLN depends on the signal-to-background ratio (SBR). To determine SBR, the Quest Research Tool (Quest Medical Imaging, Middenmeer, The Netherlands) was used. The software allows the selection of a region of interest (ROI) from the fluorescence signal of the SLN in the fluorescence image and from adjacent tissue, the background. The SBR is calculated by dividing the average intensity of the pixel values in the SLN ROI by the average intensity of the pixel values in the background. An SBR ≥ 1.1 is considered positive by NIR fluorescence. Sentinel nodes visible under normal light (Figure 1) and under fluorescence (Figure 2) were observed.

### Dilution Range to Support Findings

As our clinical research results showed, the standard concentration for injection of methylene blue (10 mg/mL) revealed a promising, but not ideal, result. We hypothesized that quenching at this high concentration might be the effect causing the suboptimal detection of fluorescence in the sentinel nodes. To support this hypothesis, ex vivo studies with the aim of finding an optimal dilution were performed, resulting in the finding that lower concentrations of methylene blue could potentially result in better visualization of the fluorescence of the dye. In future studies, the optimal dose will be one of the topics to focus on.

**Figure 3:**
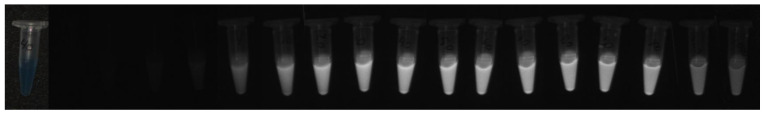
Dilution series of methylene blue visualized by fluorescence imagining device.

We conducted additional experiments to support the clinical findings. Two dilution series ranging from 0.125 to 1000 µM were created using MB stock solutions. Eppendorf vials were filled with different dilutions and the vials were imaged using a Quest Spectrum (Quest Medical Imaging, Middenmeer, The Netherlands) fluorescence imaging device, maintaining fixed camera settings, distance to the sample, and sample location (Figure 3). Analysis of the fluorescence signal in the vials was performed using the Quest Research Tool. In the fluorescence image, an ROI was selected in the sample region, and another at a fixed point in the background. Fluorescence intensities were calculated by subtracting the background intensity from the fluorescence intensity in the sample. We used MB at a basic concentration of 10 mg/mL.

## 3. Statistical Analysis

Descriptive statistics were reported either as a median with minimum and maximum values or as a frequency with percentages. Chi-squared tests were used to test for differences between observed frequencies and frequencies that were expected under the null hypothesis. A *p*-value < 0.05 was considered statistically significant. All statistical analyses were performed using the SPSS version 26.0 software package for Mac (IBM Corp., Chicago, IL, USA).

## 4. Results

This study prospectively enrolled 49 female patients with invasive breast cancer who then underwent an SLN biopsy under both radioactive and MB guidance. The fluorescence properties of MB were also used for guidance during the SLN biopsy. The median age of the patients was 62.49 years (range 42–84 years). All other clinicopathological characteristics of the study patients are listed in Table 1. Analysis of different factors associated with sentinel node biopsy are listed in Table 2. Uptake of ^99m^Tc was present in all patients. In 23 patients (46.9%), the location of the SLN, or the end of the lymphatic path, was visible transcutaneously using fluorescence. The median SBR for transcutaneous SLN location was 1.69 (range 1.66–4.35). Fluorescence in the SLN was visible in 25 patients (51%). Blue-dye-stained nodes were visible to the naked eye in 40 patients (81.6%). The median SBR for SLN visualization by fluorescence was 2.54 (range 1.34–6.86). Lymphatic channels were visible under fluorescence in 14 patients (28.6%) prior to visualization by the naked eye, with an average SBR of 2.01 (range 1.14–5.6). In three patients (6.1%), the SLN was visible under fluorescence, but not to the naked eye. In 15 patients (30.6%), the node was visible to the naked eye, but not with fluorescence visualization. We also performed analyses to test the differences between observed frequencies and frequencies that were expected under the null hypothesis between transcutaneous SLN visualization, lymphatic channel fluorescence visualization, and different clinical as well as pathological factors (Table 3). For transcutaneous SNL fluorescence visualization, statistically significant factors were smoking (*p* = 0.001) and neoadjuvant chemotherapy (*p* = 0.026). SLNs were visualized faster under fluorescence during SLN preparation. Factors associated with this included diabetes (*p* = 0.001), neoadjuvant chemotherapy (*p* = 0.003), and multifocality (*p* = 0.004). The only factor associated with visualization of the SLN by naked eye was neoadjuvant chemotherapy (*p* = 0.013).

As presented in Table 4, the optimal dilution for the highest fluorescence intensity for MB was 40 μM (0.0128 mg/mL). However, there may be potential clinical relevance for MB dilutions in the range 20 μM (0.0064 mg/mL) to 100 μM (0.032 mg/mL). We also visualized the performance of the different concentrations of MB and their corresponding fluorescence intensity (Figure 3).

## 5. Discussion

The primary endpoint of our research was the measurement of fluorescence imaging regarding the effectiveness of MB for SLN mapping in breast cancer patients. Our results revealed that in 51% of patients, we were able to visualize the fluorescence signal in the lymph nodes using the fluorescence imaging device. Based on our prior experience using ICG fluorescence during SLN biopsies for breast cancer, it appears that the performance of NIR fluorescence is comparable to that of radioactive dyes. This may lead to changes in our daily practice in the future. However, we need to further investigate why we were able to visualize fluorescence of the SLNs in only half of our study’s patients.

MB presents with different characteristics in comparison to ICG, when used as a fluorophore. By analyzing the technical differences between ICG and MB, we may be able to draw more conclusions about their functions. The main difference between the two fluorophores is the peak of visible light excitation, which presents around 700 nm for MB and 800 nm for ICG [11,14,15]. This causes MB to have a lower tissue penetration capacity, so background tissue can show more autofluorescence [11]. In the literature, the depth of penetration for ICG was determined to be between 1–1.5 cm, and for MB, up to 1 cm [24]. Additional studies are needed to examine the penetration depth of MB when employing its fluorescent properties. Both ICG and MB are currently used to identify lymph nodes, in part due to their small size. ICG has a diameter of 1.2 nm, 776 Da, and MB has a diameter of 1.43 nm, 320 Da [13]. However, their small size, especially their very small diameter, can cause these dyes to pass quickly through the lymph nodes, eventually losing the dye beyond the SLN [25,26].

The next challenge regarding the use of MB is the necessity of dimming the operative lights during surgical procedures. When ICG was first used as a fluorophore during surgery, the operative lights were dimmed during the procedure; however, advancements in technology have allowed for the use of new cameras under the normal lights used in the operating theater. This is less disturbing to the operator during surgery [13,27]. For now, dimming the operative lights is necessary when using MB, as the range of excitation and detection of MB is within the daylight spectrum. Thus, in using MB as a fluorophore, surgeons must work using only the light emitted from the camera.

### 5.1. Doses

Another problem that presents with the use of MB as a fluorophore is the determination of an optimal dose of blue dyes for an SLN biopsy. In our study, each patient received a standard dose of radiocolloid, 1 mCi in 1 mL, which was injected intradermally around the areola at a single injection point prior to the operation. The standard dose of blue dyes varies from 1–5 mL, depending on the meta-analysis. Li et al. [28] showed that dosages can vary from 0.1 mL to 10 mL. The dose of 1 mL of MB was determined as the current standard in our department.

### 5.2. Quenching

Researchers observed that a reduction in fluorescence emission occurs as the concentration of fluorophore increases [29]. From this point of view, using a high concentration of fluorophore for SLN mapping while using NIR fluorescence may prove disruptive [30]. It is probable that the dilution of NIR fluorophores may decrease the effect of quenching and affect the NIR signal of the dye. Another concern is that the high concentration of MB in the sentinel lymph node may be responsible for the difficulties in fluorescence visualization of these structures using NIR light. This phenomenon may play a role in the observations in our study’s group of patients. We hypothesized that lower doses of MB may improve our visualization. In our preclinical study, we found that, with lower concentration, the fluorescence of MB increased, reaching its peak at 40 μM (0.0128 mg/mL). In our analysis of different dilutions for the highest fluorescence intensity, it seemed that dilution might be key to improving the intensity of fluorescence; thus, a future study should focus on this idea to show the optimal dilution in a clinical setting, which may be different from the laboratory value presented in our study.

### 5.3. Safety Profile and Side Effects

MB has been used in the field of surgery for decades, typically presenting with a good safety profile [3,19,31,32,33,34]. Skin necrosis has been reported, as well as fat and parenchymal necrosis [35,36,37]. Other skin reactions were also reported by Kaklamanos et al. [38]. In some patients, skin discoloration may be seen after blue dye injection, which can persist as a tattoo for up to one year after surgery [39]. Persistent skin discoloration is also observed after the use of other fluorophores such as ICG for SLN biopsy in breast cancer [40]. In our study, no adverse reactions or events were noted by our patients.

### 5.4. Identification Rate

The identification rate of SNB when using only blue dye is about 91%. We can improve the detection rate by performing SNB using a second dye with radioactive properties, especially nanocolloids. With this double dye technique, detection rates improved to 96% [5]. When ICG was used, sentinel lymph nodes could be identified in breast cancer patients by the naked eye 50% of the time. When using ICG’s fluorescent properties, SLN could be visualized 94% of the time [6]. In our study, SLNs were stained blue during surgery in 81.6% of patients (40/49). Using a near-infrared fluorescence imaging camera system, we were able to visualize fluorescence in the SLNs with MB in 51% of cases (25/49). Interestingly, in three patients (7%), SLNs were seen only with MB fluorescence. We must underline the fact that in 17 patients (37%), the SLNs were seen only with the naked eye, but were not visible under fluorescence.

It appears that the visualization of MB under fluorescence is the reverse of ICG, where identification of SLN using the naked eye is much lower than when using its fluorescent properties. According to Motomura et al., in a group of 172 patients, SLNs stained with ICG were visualized by the naked eye in 73.8% of the patients [41].

The low rate of fluorescent lymph nodes when using MB is likely due to the quenching effect phenomenon. Similar problems were identified after initial studies using ICG for SNB were published. In a study by Mieog et al., researchers found that, with an increase in concentration, the quenching effect disturbed the visualization of ICG-stained structures. They recommended a dose of 0.62 mg of ICG as an optimal dose for fluorescence navigation [30]. Other papers reported the use of doses of ICG between 0.625 mg and 15 mg in a volume of between 1 and 5 mL respectively, achieving similar detection rates [42]. We presented an in vitro analysis of different dilutions of MB, which showed that its fluorescence intensity reached its peak with a dilution of 40 μM (0.0128 mg/mL), beyond which the fluorescence intensity became much smaller. Thus, further research is needed to determine the optimal dose of MB in a clinical setting when using its fluorescent properties.

The most important advantage of MB is its long-term clinical use and well-documented experience by many surgeons. We must point out that a low-detection rate of SLN under fluorescence guidance is a critical point of our study. Future technological advancements, the use of new cameras, or the possible use of other blue dyes as fluorophores may all be solutions to this dilemma.

## 6. Conclusions

Our study presents the possibility of performing SNB using an old dye in a new fashion with fluorescence visualization. The standard dose of MB did not seem to be sensitive enough in fluorescence visualization of SLNs in breast cancer. However, we suppose that MB as a fluorophore may prove to be a useful tool in the future, especially at lower concentrations. As the in vitro dilution test represents samples that have only been diluted and not injected into the patient, it should be considered that the concentration in the SLN may be lower than the concentration injected. Therefore, further in vivo investigation is needed to indicate the optimal injection concentration for the best fluorescence visualization of SLNs using MB.

## Figures and Tables

**Table 1:** Clinicopathological characteristic of 49 enrolled patients.

Variable	Category	N. Cases (%)
All patients		49 (100)
Age, years [median (min-max)]		62 (42–84)
BMI [median (min-max)]		28 (17–52)
Diabetes	No	47 (95.9)
	Yes	2 (4.1)
Smoking	No	34 (69.4)
	Yes	15 (30.6)
Tumor localization	Upper outer	29 (59.2)
	Upper medial	8 (16.3)
	Lower outer	8 (16.3)
	Lower medial	4 (8.2)
Multifocality	No	41 (83.7)
	Yes	8 (16.3)
Neoadjuvant chemotherapy	No	43 (87.8)
	Yes	6 (12.2)
Neoadjuvant hormone therapy	No	46 (93.9)
	Yes	3 (6.1)
Histological grade	I	18 (36.7)
	II	19 (38.8)
	III	11 (22.4)
	Missing	1 (2.0)
Molecular type	Luminal A	21 (41.9)
	Luminal B	10 (20.4)
	Luminal HER2+	7 (14.3)
	Non-luminal HER2+	2 (4.1)
	Triple negative	8 (16.3)
	Missing	1 (2.0)
Estrogen	No	10 (20.4)
	Yes	38 (77.6)
	Missing	1 (2.0)
Progesterone	No	14 (28.6)
	Yes	34 (69.4)
	Missing	1 (2.0)
HER2	No	36 (73.5)
	Yes	12 (24.5)
	Missing	1 (2.0)
Ki-67 [median (min-max)]		10 (1–85)

**Table 2:** Analysis of sentinel lymph node data.

Variable	Category	Number of Cases (%)
Number of sentinel nodes	1	26 (53.1)
	2	14 (28.6)
	3	4 (8.2)
	4	3 (6.2)
	5	1 (2.0)
	Missing	1 (2.0)
Type of metastatic nodes	Missing	1 (2.0)
	No metastasis	30 (61.2)
	Micrometastasis	1 (2.0)
	Macrometastasis	14 (28.6)
	Isolated cancer cells	2 (4.1)
Radiocolloid nodes stained	Missing	1 (2.0)
	Negative	0
	Positive	49
NIRF visibility through skin	Negative	26 (53.1)
	Positive	23 (46.9)
NIRF visibility of nodes	Negative	24 (49)
	Positive	25 (51)
Blue nodes stained (eye)	Negative	9 (18.4)
	Positive	40 (81.6)
NIRF visibility of lymphatic vessels	Negative	35 (71.4)
	Positive	14 (28.6)

NIRF, near-infrared fluorescence.

**Table 3:** Critical values of the Chi-Square test for factors associated with near-infrared fluorescence visibility through skin and of sentinel lymph nodes in comparison to naked eye visibility of blue-stained nodes.

Variable	Category	NIRF Visibility through Skin		(*p* Value)	NIRF Visibility of Nodes		(*p* Value)	Blue Nodes Stained (Eye)		(*p* Value)
		0	1		0	1		0	1	
Diabetes	No	24	23	1.84	23	24	0.001	8	39	1.39
	Yes	2	0		1	1		1	1	
Smoking	No	18	16	0.001	17	17	0.046	7	27	0.365
	Yes	8	7		7	8		2	13	
Neoadjuvant chemotherapy	No	23	20	0.026	21	22	0.003	8	35	0.013
	Yes	3	3		3	3		1	5	
Neoadjuvant hormone therapy *	No	24	21	0.201	22	23	0.356	7	38	4.82
	Yes	2	1		2	1		2	1	
Multifocality	No	24	17	3.02	20	21	0.004	7	34	0.281
	Yes	2	6		4	4		2	6	
Molecular type *	Luminal A	11	10	0.762	10	11	3.73	4	17	3.11
	Luminal B	6	4		6	4		3	7	
	Luminal HER2+	3	4		2	5		0	7	
	Non-luminal HER2+	1	1		2	0		0	2	
	Triple negative	5	3		4	4		2	6	
Histological grade *	I	12	6	2.25	9	9	1.29	3	15	1.44
	II	8	11		11	8		5	14	
	III	6	5		4	7		1	10	
NIRF_lymphatic channel	0	19	16	0.074	10	25	20.4	8	27	1.65
	1	7	7		14	0		1	13	

NIRF, near-infrared fluorescence. * Values are given according to number of patients per group after exclusion of patients with potential missing data.

**Table 4:** Fluorescence intensity in different Methylene Blue dilutions.

uM	mg/mL	Average Intensity	Standard Deviation
0.125	0.00004	8.8	1.7
0.25	0.00008	9.8	1.8
0.5	0.00016	13	2
1	0.00032	17.2	2.3
4	0.00128	60	4.8
10	0.0032	106.6	8.2
20	0.0064	140.3	8.4
30	0.0096	159.1	8.6
40	0.0128	164.4	7.4
50	0.016	162.4	7.2
60	0.0192	157.3	7
70	0.0224	157.7	6.2
80	0.0256	156.6	6.9
90	0.0288	146.1	5.8
100	0.032	141.8	5.5
500	0.16	79.3	4.1
1000	0.32	59.5	3.7

## Data Availability

The data presented in this study are available on request from the corresponding author.

## References

[1] Veronesi U., Viale G., Paganelli G., Zurrida S., Luini A., Galimberti V., Veronesi P., Intra M., Maisonneuve P., Zucca P. (2010). Sentinel Lymph Node Biopsy in Breast Cancer: Ten-Year Results of a Randomized Controlled Study. Ann. Surg..

[2] Zahoor S., Haji A., Battoo A., Qurieshi M., Mir W., Shah M. (2017). Sentinel Lymph Node Biopsy in Breast Cancer: A Clinical Review and Update. J. Breast Cancer.

[3] Morton D.L., Wen D.R., Wong J.H., Economou J.S., Cagle L.A., Storm F.K., Foshag L.J., Cochran A.J. (1992). Technical Details of Intraoperative Lymphatic Mapping for Early Stage Melanoma. Arch. Surg..

[4] Giuliano A.E., Kirgan D.M., Guenther J.M., Morton D.L. (1994). Lymphatic Mapping and Sentinel Lymphadenectomy for Breast Cancer. Ann. Surg..

[5] Niebling M.G., Pleijhuis R.G., Bastiaannet E., Brouwers A.H., Van Dam G.M., Hoekstra H.J. (2016). A Systematic Review and Meta-Analyses of Sentinel Lymph Node Identification in Breast Cancer and Melanoma, A Plea for Tracer Mapping. Eur. J. Surg. Oncol..

[6] Kitai T., Inomoto T., Miwa M., Shikayama T. (2005). Fluorescence Navigation with Indocyanine Green for Detecting Sentinel Lymph Nodes in Breast Cancer. Breast Cancer.

[7] Zhang C., Jiang D., Huang B., Wang C., Zhao L., Xie X., Zhang Z., Wang K., Tian J., Luo Y. (2019). Methylene Blue-Based Near-Infrared Fluorescence Imaging for Breast Cancer Visualization in Resected Human Tissues. Technol. Cancer Res. Treat..

[8] Liu J., Huang L., Wang N., Chen P. (2017). Indocyanine Green Detects Sentinel Lymph Nodes in Early Breast Cancer. J. Int. Med. Res..

[9] Chi C., Ye J., Ding H., He D., Huang W., Zhang G.-J., Tian J. (2013). Use of Indocyanine Green for Detecting the Sentinel Lymph Node in Breast Cancer Patients: From Preclinical Evaluation to Clinical Validation. PLoS ONE.

[10] Vahrmeijer A.L., Hutteman M., Van Der Vorst J.R., Van De Velde C.J.H., Frangioni J.V. (2013). Image-Guided Cancer Surgery Using Near-Infrared Fluorescence. Nat. Rev. Clin. Oncol..

[11] van Manen L., Handgraaf H.J.M., Diana M., Diijkstra J., Ishizawa T., Vahrmeijer A.L., Mieog J.S.D. (2018). A Practical Guide for The Use of Indocyanine Green and Methylene Blue in Fluorescence-Guided Abdominal Surgery. J. Surg. Oncol..

[12] Schaafsma B.E., Mieog J.S.D., Hutteman M., Van der Vorst J.R., Kuppen P.J.K., Lowik C.W.G.M., Frangioni J.V., Van de Velde C.J.H., Vahrmeijer A.L. (2011). The Clinical Use of Indocyanine Green as A Near-Infrared Fluorescent Contrast Agent for Image-Guided Oncologic Surgery. J. Surg. Oncol..

[13] Polom K., Murawa D., Rho Y.S., Nowaczyk P., Hünerbein M., Murawa P. (2011). Current Trends and Emerging Future of indocyanine Green Usage in Surgery and Oncology: A Literature Review. Cancer.

[14] Ginimuge P.R., Jyothi S.D. (2010). Methylene Blue: Revisited. J. Anaesthesiol. Clin. Pharmacol..

[15] Barnes T.G., Hompes R., Birks J., Mortensen N.J., Jones O., Lindsey I., Guy R., George B., Cunningham C., Yeung T.M. (2018). Methylene Blue Fluorescence of The Ureter During Colorectal Surgery. Surg. Endosc..

[16] Stummer W., Pichlmeier U., Meinel T., Wiestler O.D., Zanella F., Reulen H.J. (2006). Fluorescence-Guided Surgery with 5-Aminolevulinic Acid for Resection of Malignant Glioma: A Randomised Controlled Multicentre Phase III Trial. Lancet Oncol..

[17] Verbeek F.P.R., Van Der Vorst J.R., Schaafsma B.E., Swijnenburg R.-J., Gaarenstroom K.N., Elzavier H.W., van de Velde C.J.H., Frangioni J.V., Vahrmeijier A.L. (2013). Intraoperative Near Infrared Fluorescence Guided Identification of the Ureters Using Low Dose Methylene Blue: A First in Human Experience. J. Urol..

[18] Al-Taher M., Van Den Bos J., Schols R.M., Bouvy N.D., Stassen L.P.S. (2016). Fluorescence Ureteral Visualization in Human Laparoscopic Colorectal Surgery Using Methylene Blue. J. Laparoendosc. Adv. Surg. Tech..

[19] Hillary S.L., Guillermet S., Brown N.J., Balasubramanian S.P. (2018). Use of Methylene Blue and Near-Infrared Fluorescence in Thyroid and Parathyroid Surgery. Langenbeck’s Arch. Surg..

[20] McWade M.A., Thomas G., Nguyen J.Q., Sanders M.E., Solórzano C.C., Mahadevan-Jansen A. (2019). Enhancing Parathyroid Gland Visualization Using a Near Infrared Fluorescence-Based Overlay Imaging System. J. Am. Coll. Surg..

[21] De Leeuw F., Breuskin I., Abbaci M., Casiraghi O., Mirghani H., Lakhdar A.B., Laplace-Builhe C., Hartl D. (2016). Intraoperative Near-Infrared Imaging for Parathyroid Gland Identification by Auto-Fluorescence: A Feasibility Study. World J. Surg..

[22] Paras C., Keller M., White L., Phay J., Mahadevan-Jansen A. (2011). Near-Infrared Autofluorescence for the Detection of Parathyroid Glands. J. Biomed. Opt..

[23] Winer H.J., Choi S.H., Gibbs-Strauss L.S., Ashitate Y., Colson L.Y., Frangioni V.J. (2010). Intraoperative Localization of insulinoma and Normal Pancreas Using Invisible Near-Infrared Fluorescent Light. Ann. Surg. Oncol..

[24] Teraphongphom N., Kong C.S., Warram J.M., Rosenthal E.L. (2017). Specimen Mapping in Head and Neck Cancer Using Fluorescence Imaging. Laryngosc. Investig. Otolaryngol..

[25] Cousins A., Thompson S.K., Wedding A.B., Thierry B. (2014). Clinical Relevance of Novel Imaging Technologies for Sentinel Lymph Node Identification and Staging. Biotechnol. Adv..

[26] Mieog J.S.D., Hutteman M., van der Vorst J.R., Kuppen P.J.K., Que I., Dijkstra J., Kaijzel E.L., Prins F., Lowik C.W.G.M., Smit V.T.H.B.M. (2011). Image-Guided Tumor Resection Using Real-Time Near-Infrared Fluorescence in a Syngeneic Rat Model of Primary Breast Cancer. Breast Cancer Res. Treat..

[27] van den Berg N.S., Miwa M., KleinJan G.H., Maeda Y., van Akkooi A.C.J., Horenblas S., Karakullukcu B., van Leeuwen F.W.B. (2016). (Near-Infrared) Fluorescence-Guided Surgery under Ambient Light Conditions: A Next Step to Embedment of the Technology in Clinical Routine. Ann. Surg. Oncol..

[28] Li J., Chen X., Qi M., Li Y. (2018). Sentinel lymph node biopsy mapped with methylene blue dye alone in patients with breast cancer: A systematic review and meta-analysis. PLoS ONE.

[29] Luo T., Zhou T., Zhao Y., Liu L., Qu J. (2018). Multiplexed Fluorescence Lifetime Imaging by Concentration-Dependent Quenching. J. Mater. Chem. B.

[30] Mieog J.S.D., Troyan S.L., Hutteman M., Donohoe K.J., van der Vorst J.R., Stockdale A., Liefers G.-J., Choi H.S., Gibbs-Strauss S.L., Putter H. (2011). Toward Optimization of Imaging System and Lymphatic Tracer for Near-Infrared Fluorescent Sentinel Lymph Node Mapping in Breast Cancer. Ann. Surg. Oncol..

[31] Gould E.A., Winship T., Philbin P.H., Kerr H.H. (1960). Observations on a “Sentinel Node” in Cancer of the Parotid. Cancer.

[32] Cabanas R.M. (1977). An Approach for the Treatment of Penile Carcinoma. Cancer.

[33] Chung A., Giuliano A.E. (2018). Lymphatic Mapping and Sentinel Lymphadenectomy for Breast Cancer. Breast Compr. Manag. Benign Malig. Dis..

[34] Mulsow J., Winter D.C., O’Keane J.C., O’Connell P.R. (2003). Sentinel Lymph Node Mapping in Colorectal Cancer. Br. J. Surg..

[35] Stradling B., Aranha G., Gabram S. (2002). Adverse Skin Lesions After Methylene Blue Injections for Sentinel Lymph Node Localization. Am. J. Surg..

[36] Salhab M., Al Sarakbi W., Mokbel K. (2005). Skin and Fat Necrosis of the Breast Following Methylene Blue Dye Injection for Sentinel Node Biopsy in a Patient with Breast Cancer. Int. Semin. Surg. Oncol..

[37] Reyes F.J., Noelck M.B., Valentino C., Grasso-LeBeau L., Lang J.E. (2011). Complications of Methylene Blue Dye in Breast Surgery: Case Reports and Review of the Literature. J. Cancer.

[38] Kaklamanos I.G., Birbas K., Syrigos K., Bonatsos V.G., Bonatsos G. (2011). Prospective Comparison of Peritumoral and Subareolar Injection of Blue Dye Alone, for Identification of Sentinel Lymph Nodes in Patients with Early Stage Breast Cancer. J. Surg. Oncol..

[39] Borgstein P.J., Meijer S., Pijpers R. (1997). Intradermal Blue Dye to Identify Sentinel Lymphnode in Breast Cancer. Lancet.

[40] Murawa D., Polom K., Murawa P. (2014). One-Year Postoperative Morbidity Associated with Near-Infrared-Guided Indocyanine Green (ICG) or ICG in Conjugation with Human Serum Albumin (ICG: HSA) Sentinel Lymph Node Biopsy. Surg. Innov..

[41] Motomura K., Inaji H., Komoike Y., Kasugai T., Noguchi S., Koyama H. (1999). Sentinel Node Biopsy Guided by Indocyanine Green Dye in Breast Cancer Patients. Jpn. J. Clin. Oncol..

[42] Ahmed M., Purushotham A.D., Douek M. (2014). Novel Techniques for Sentinel Lymph Node Biopsy in Breast Cancer: A Systematic Review. Lancet Oncol..

